# Editorial Special issue: *Valorization of Plant-Based Products by Bio-Technological and Green Extraction Approaches: Recent Advances and Applications*^☆^

**DOI:** 10.1016/j.fochx.2026.103850

**Published:** 2026-04-09

**Authors:** Angela Cardinali, Gianluca Bleve

**Affiliations:** a Consiglio Nazionale delle Ricerche - Istituto di Scienze delle Produzioni Alimentari, Bari, 70126 Bari, Italy; b Consiglio Nazionale delle Ricerche - Istituto di Scienze delle Produzioni Alimentari, Unità Operativa di Lecce, 73100 Lecce, Italy

The increasing global demand for healthier food products, sustainable production systems, and the efficient use of natural resources has driven substantial research efforts toward functional bioactive compounds, advanced extraction technologies, and environmentally sustainable processing strategies. The agri-food sector generates considerable amounts of co/by-products, many of which remain underutilized and are often managed as waste, resulting in environmental and economic inefficiencies. However, these co/by-products frequently contain high-value bioactive compounds that can be recovered and exploited for the development of novel ingredients and products with applications across multiple industrial sectors.

This Special Issue compiles multidisciplinary research addressing the characterization of **bioactive compounds, fermentation-based processes, green extraction technologies, agro-industrial by-product valorisation, and functional food development**. The contributions collectively aim to advance current understanding of sustainable agri-food co-product utilization and to highlight their potential roles in food, nutrition, health, cosmetics, and environmental sustainability. In total, 77 manuscripts were submitted, of which 35 were accepted for publication, all addressing the management and valorization of agro-industrial co- and by-products. The studies encompass a wide range of plant-based matrices, including fruits, flowers, roots, seeds, mushrooms, coffee, as well as food products such as fruit juice and wines, but also seaweed and microalgae, reflecting the broad scope and relevance of this field.

## 1 **Bioactive Compounds and Health-Promoting Functions**

Several contributions focused on identifying and evaluating bioactive compounds with antioxidant, anti-inflammatory, metabolic-regulating, and immune-modulating properties deriving from a plethora of plants and animals by-products. The carotenoid astaxanthin from marine organisms was comprehensively reviewed for its pigmentation, antioxidant, immune-enhancing, and commercial value in aquaculture and for its applications in medicine and cosmetics (Zhu, Li, & Wu, 2025). Interestingly, a new potential use for cyanobacterial species *Microcystis flos-aquae* has been proposed as a source for astaxanthin production, and KCl stress was identified as the main factor inducing the compound production (Ma et al., 2025).

In another study, guava (*Psidium guajava*) was reviewed as a multifunctional medicinal plant rich in flavonoids with anti-inflammatory, antidiabetic, antihypertensive, and antioxidant activities, supporting its use in functional foods (Butt et al., 2025). Polyphenol-rich extracts with strong antioxidant, anti-inflammatory, and hypoglycemic activities were obtained by *Phyllanthus emblica* L. fruit using enzyme-assisted extraction (Qu, Wang et al., 2025). A therapeutic potential against allergic inflammatory diseases was demonstrated for cashew nut oligopeptides, which inhibit mast cell degranulation through suppression of the NF-κB signalling pathway (Chen, Zhou, Zhang, & Chen, 2025). Camellia seed cake extract, rich in flavonoids, ameliorated glycolipid metabolism disorders in diabetic mice via Acyl-CoA oxidase 1 (ACOX1) inhibition (B. Chen et al., 2025). Moreover, artificial fermentation of dark loose tea improved metabolic-associated fatty liver disease by activating the PI3K/AKT pathway and modulating gut microbiota composition (Yuan et al., 2025).

## 2 **Pigments, Polyphenols, and Antioxidant Systems**

Natural pigments and phenolic compounds were a recurring theme. The pigment composition and antioxidant capacity of seven colored pummelo cultivars are systematically analyzed, revealing strong correlations between carotenoids, anthocyanins, flavonoids, and antioxidant activity (Zhang et al., 2025). A comprehensive evaluation of the physicochemical properties, composition profile, and genes related to carotenoid and fatty acid biosynthesis of *Stauntonia obovatifoliola* seed oil highlighted its potential applications in the food, cosmetic, and pharmaceutical industries (Li, Zhong, Zhou, Wang, & Huang 2025). In addition, durian flowers, an underutilized agricultural waste, were identified as a novel source of procyanidins with antioxidant and anti-inflammatory effects, and the *Amomum tsaoko* medicinal and food plant rich in flavonoids, revealed their potential in nutraceutical and cosmeceutical applications (Huang et al., 2025; Potijun et al., 2025).

## 3 **Fermentation and Microbial Biotechnology**

Fermentation emerged as a powerful strategy to enhance nutritional quality, bioavailability, and sensory attributes of wines from traditional (Xie et al., 2024) or innovative sources (Chen, Lei, Sun et al., 2025). A special attention was addressed to the effects of high ethanol levels to the quality of industrial Cabernet Sauvignon wines through aging in oak barrels (Chen, Lei, Zang et al., 2025). In addition, *Lactobacillus plantarum* fermentation improves the flavor, acceptability, and metabolomic profile of *Aronia melanocarpa* elliott juice (Liu et al., 2025), while *Saccharomyces cerevisiae* fermentation modulates non-volatile metabolites and sensory quality in Robusta coffee beans (Yu, Huang, Fu, Hu, & Dong 2025) A probiotic beverage developed from fermented hibiscus tea and coconut water exhibited strong antioxidant and probiotic properties (Aher et al., 2025). Solid-state fermentation driven by *Aspergillus awamori* (MTCC 548) enhanced functional properties, mineral bioavailability, and digestibility of kidney bean flour (Patil et al., 2025), whereas fermented soybean meal was verified as a good supplement to improve soil health, rhizosphere microbial communities, and tea quality, reinforcing the role of fermentation in sustainable agriculture (Wang et al., 2025).

## 4 **Green Extraction Technologies and Sustainable Solvents**

Several contributes of this Special Issue was devoted to green and efficient extraction methodologies. Supercritical CO₂ extraction of hemp seed oil reveals how processing parameters affect oil composition and bioactivity (Vishwasrao, Zengin, Sinan, Minceva, & Luca, 2025). Several studies were addressed to protein extraction methods from several sources. Deep eutectic solvents were applied for extracting functional proteins and bioactive peptides from *Torreya grandis* and mushroom stem agro-waste, demonstrating improved sustainability and functionality compared to conventional methods (Durrani et al., 2025; Karabulut*,* Köroğlu, Feng & Karabulut, 2024). A combination of ultrasound-assisted extraction (UAE) and alkaline extraction (AE) methods were successfully applied to defatted pequi almond cake for producing protein isolates (Ribeiro et al., 2025).

A glabridin (GLA)-rich extract from licorice with interesting antimicrobial activities was obtained developing environmentally sustainable ultrasonic-assisted aqueous two-phase extraction method (Qu, Zhu et al., 2025). Alternative bio-based solvents such as 2-methyloxolane were proposed as green replacements for n-hexane in camellia oil extraction (Lin, Wang, & Li, 2025), while aqueous two-phase systems based on DES were developed for flavonoid extraction and detection in honey (Zhang et al., 2024). Moreover, *Citrus aurantium* flower extracts obtained via sequential green extraction demonstrated strong antioxidant efficiency and improve oil oxidative stability when encapsulated (Kellil et al., 2025). Deng et al. (2024) optimized the extraction conditions and the harvesting periods suitable to maximize the flavonoid glycoside content of edible *Dendrobium officinale* leaves*.*

## 5 **Valorization of Agro-Waste and Circular Bioeconomy**

Valorization of food and agricultural by-products was a core subject. Pea peel waste was reviewed for its transformation into polyphenol extracts, dietary fiber, biodegradable films, biosorbents, and biochar, contributing to a circular bioeconomy (Agarwal et al., 2025). Spent coffee grounds were successfully converted into D-mannose and bioethanol through integrated pretreatment, enzymatic hydrolysis, and selective fermentation (Cho et al., 2025), while apple pomace was valorized through green extraction of low-methoxyl pectins characterized by desirable rheological and gelling properties for low-calorie food products (Guo et al., 2024).

Finally, an extensive review on formation mechanism, influencing factors, functional properties and food applications of insoluble protein aggregates was provided (Ji et al., 2025).

## 6 **Functional Foods, Packaging, and Product Innovation**

Several studies explored the optimization or *de novo* development of novel foods. Microwave-assisted extraction enhanced Aquafaba functionality as a sustainable egg-white replacer in vegan meringues (Kargar and Sourki (2025). The potential of seaweed-based functional foods was demonstrated by the optimization of *Gracilaria lemaneiformis* jelly using fuzzy mathematics (Hu & Wu, 2025). Active antibacterial films based on tapioca starch and clove essential oil offered biodegradable packaging solutions that extended bread shelf life (Chang, Zhao, Zhang, Chen, & Yang, 2025).

## 7 **Conclusions and outlook**

Collectively, the studies in this Special Issue underscore the **synergistic role of bioactive compound discovery, green processing technologies, fermentation, and waste valorization** in advancing sustainable food systems. By bridging fundamental research with applied innovation, these contributions provide valuable insights and practical solutions for the development of functional foods, nutraceuticals, eco-friendly materials, and circular bioeconomy strategies. This Special Issue thus offers a comprehensive reference for researchers, industry professionals, and policymakers working toward healthier and more sustainable food futures.
